# Altered Organelle Calcium Transport in Ovarian Physiology and Cancer

**DOI:** 10.3390/cancers12082232

**Published:** 2020-08-10

**Authors:** Laura Caravia, Cristina Elena Staicu, Beatrice Mihaela Radu, Carmen Elena Condrat, Dragoș Crețoiu, Nicolae Bacalbașa, Nicolae Suciu, Sanda Maria Crețoiu, Silviu Cristian Voinea

**Affiliations:** 1 Department of Cell and Molecular Biology and Histology, Carol Davila University of Medicine and Pharmacy, 050474 Bucharest, Romania; lauracaravia76@gmail.com (L.C.); dragos@cretoiu.ro (D.C.); 2 Department of Anatomy, Animal Physiology and Biophysics, Faculty of Biology, University of Bucharest, Splaiul Independentei 91-95, 050095 Bucharest, Romania; elena.necsulescu@drd.unibuc.ro (C.E.S.); beatrice.radu@bio.unibuc.ro (B.M.R.); 3 Center for Advanced Laser Technologies (CETAL), National Institute for Laser, Plasma and Radiation Physics, 409 Atomiștilor St., 77125 Măgurele, Romania; 4 Life, Environmental and Earth Sciences Division, Research Institute of the University of Bucharest (ICUB), 91-95 Splaiul Independenţei, 050095 Bucharest, Romania; 5 Alessandrescu-Rusescu National Institute of Mother and Child Health, Fetal Medicine Excellence Research Center, 020395 Bucharest, Romania; drcarmencondrat@gmail.com (C.E.C.); nsuciu54@yahoo.com (N.S.); 6 Department of Obstetrics and Gynecology, Carol Davila University of Medicine and Pharmacy, 050474 Bucharest, Romania; nicolae_bacalbasa@yahoo.ro; 7 Department of Surgical Oncology, Prof. Dr. Alexandru Trestioreanu Oncology Institute, Carol Davila University of Medicine and Pharmacy, 252 Fundeni Rd., 022328 Bucharest, Romania; dr.voineasilviu@gmail.com

**Keywords:** ovarian cancer, organelles, calcium signaling, drug resistance

## Abstract

Calcium levels have a huge impact on the physiology of the female reproductive system, in particular, of the ovaries. Cytosolic calcium levels are influenced by regulatory proteins (i.e., ion channels and pumps) localized in the plasmalemma and/or in the endomembranes of membrane-bound organelles. Imbalances between plasma membrane and organelle-based mechanisms for calcium regulation in different ovarian cell subtypes are contributing to ovarian pathologies, including ovarian cancer. In this review, we focused our attention on altered calcium transport and its role as a contributor to tumor progression in ovarian cancer. The most important proteins described as contributing to ovarian cancer progression are inositol trisphosphate receptors, ryanodine receptors, transient receptor potential channels, calcium ATPases, hormone receptors, G-protein-coupled receptors, and/or mitochondrial calcium uniporters. The involvement of mitochondrial and/or endoplasmic reticulum calcium imbalance in the development of resistance to chemotherapeutic drugs in ovarian cancer is also discussed, since Ca^2+^ channels and/or pumps are nowadays regarded as potential therapeutic targets and are even correlated with prognosis.

## 1. Introduction

The mutations that lead to the development of cancer cells produce changes in many signaling processes, as new bioinformatics studies have shown [1,2]. In fact, it has become more and more evident that the signaling pathways responsible for tumor cell growth and survival point in the same direction, namely, toward cellular metabolism [3]. The reprogramming of energy metabolism and the resulting hindrance of mitochondrial function are considered a hallmark for malignant transformation [4]. In recent years, novel theories involving oncogenic mechanisms that alter calcium signaling have begun to emerge. Oncogenic alterations in calcium signaling promote cancer cell survival mainly by inhibiting apoptosis [5].

**Figure 1:**
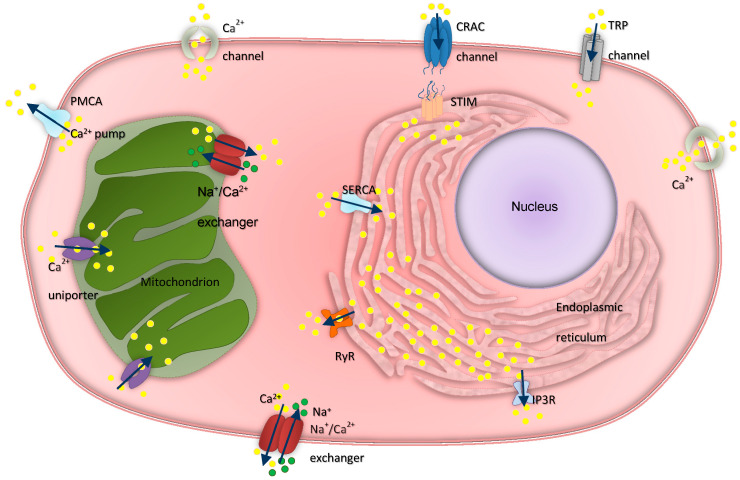
Calcium homeostasis. Ca^2+^ channels, pumps, and exchangers act in synergy to maintain normal calcium concentrations. PMCA = plasma membrane Ca^2+^-ATPase, CRAC = calcium release-activated channel, TRP = transient receptor potential channel, IP3R = inositol trisphosphate receptor, RyR = ryanodine receptor, SERCA = sarco-/endoplasmic reticulum Ca^2+^-ATPase, PMCA = plasma membrane Ca^2+^-ATPase.

Calcium ions play significant roles in numerous cellular activities, their intracellular concentration affecting nearly every cellular process, from energy output regulation and cellular metabolism to phenotype development [6]. Regulation of cytoplasmic calcium concentration depends on how Ca^2+^ is actively pumped from the cytosol to the extracellular space, and on how it enters the cell through the plasma membrane [7]. There are three major classes of membrane-associated proteins that render these processes possible: (a) channels, (b) pumps (ATPases), and (c) exchangers [8], which are briefly illustrated in Figure 1.

Although most often used synonymously, voltage-gated calcium channels (VGCCs) are actually a subtype of calcium channels, along with ligand-gated calcium channels [9]. While the former are opened through the depolarization that occurs as the result of an increase in membrane potential [10], the latter are dependent on the attachment of a binding agent to the receptor [11]. Channels consist of a single gate ensuring the passive flow of ions in a downhill manner, whereas pumps have two gates alternately closing and opening, which are connected to an energy source, thus ensuring an active form of ion transport [12]. Exchangers, on the other hand, make up a different type of active transport, involving the use of one ion’s gradient in order to guide the transfer of another [13].

Ca^2+^-binding proteins in organelles act as buffers in the release/storage of Ca^2+^ from/to intracellular deposits, e.g., endoplasmic reticulum (ER), nucleus, and mitochondria, while also participating in its homeostasis [14]. An unusual distribution of Ca^2+^ represents the basis for many diseases [15], ranging from pathologic conditions of the nervous system [16], including Alzheimer’s [17,18], to various types of arrhythmias [19,20], skin disorders [21], as well as different forms of cancer [22,23,24]. T lymphocytes, for instance, being some of the best characterized cells regarding the role of calcium in cell fate determination, are greatly influenced by intracellular Ca^2+^ [25]. Their concentration is rigorously governed by Ca^2+^ release-activated channels (CRACs) and the store-operated calcium entry (SOCE) mechanism, which are, in turn, regulated by stromal interaction molecule 1 (STIM1) [26]. Defective calcium entry due to dysfunctional CRAC channels has been shown to be associated with T-cell inactivation, resulting in severe combined immunodeficiency disorders [27,28]. Furthermore, cell proliferation is also closely related to calcium influx. The remodeling of calcium homeostasis has been reported in cancerous cells, which are characterized by their ability to proliferate in media lacking Ca^2+^ [29]. Nowadays, there are numerous data showing that cancer progression might be due, in part, to the overexpression and/or aberrant activation of Ca^2+^-specific channels and Ca^2+^-regulated intracellular pathways [22,30]. To this extent, in prostate cancer cells, Prevarskaya et al. have observed that Ca^2+^ influx through transient receptor potential channels (TRPs) can lead to an increase in angiogenic and mitogenic factors [31].

On the other hand, it has been indicated that calcium dyshomeostasis can also result in cancer cell death, with Pajak et al. highlighting that colon adenocarcinoma cells are susceptible to apoptosis as a result of intracellular calcium decreases [32,33]. Furthermore, Høyer-Hansen et al. have shown that elevations of cytosolic calcium induced by agents such as vitamin D, ionomycin, and thapsigargin can trigger autophagy through the Ca^2+^/CaMKKβ/AMPK/mTOR pathway in breast cancer cells [32]. Further on, there is increasing evidence that mitochondrial calcium is essential for the fate of the cell, since calcium overload might serve as a pro-apoptotic player by favoring the release of mitochondrial apoptotic factors into the cytosol and triggering programmed cell death [33].

The interaction between mitochondria and the ER―the most important intracellular Ca^2+^ store―in response to changes in the cellular metabolism has long been demonstrated [34,35,36]. The mitochondria–ER interaction has been physically isolated [37] and described as mitochondria-associated ER membranes establishing a distinct microdomain with specific signaling functions [38]. At these contact sites, Ca^2+^ is transferred from the ER to the mitochondria while also enabling mitochondrial trafficking, lipid synthesis and transfer, apoptosis, autophagy, and protein homeostasis, all of which are frequently altered in oncogenesis and cancer [39].

Calcium signaling alterations [40,41,42] or signaling pathway network alterations [1,43] in ovarian cancer have previously been analyzed, with most studies focusing on calcium signaling pathways triggered by ion channels/receptors/pumps from the plasma membrane. However, a systematic overview of calcium signaling alterations in organelles in ovarian cancer is largely missing. In this paper, we review the role of the main players localized in intracellular organelles that are regulating intracellular calcium (e.g., inositol trisphosphate receptors, ryanodine receptors, transient receptor potential channels, calcium ATPases, hormone receptors, mitochondrial Ca^2+^ channels) in the major components of the ovary. Here, we provide an integrated overview of the importance of calcium regulation in ovarian physiology and how altered organelle calcium transport contributes to oncogenic alterations and tumor progression, invasion, and metastasis in ovarian cancer.

## 2. Understanding Ovarian Cancer

As the primary female reproductive organs, the ovaries are responsible for oogenesis along with production and secretion of the female sex hormones. Shielded within the peritoneal cavity, the ovary is covered by the germinal epithelium, a single layer of cuboidal cells that turn into squamous cells with age [44]. It is comprised of a well-established aggregate of oocytes and somatic cells, including theca cells, which are responsible for androgen production, acting as substrate for estrogen synthesis [45]; granulosa or follicular cells, which are in charge of aromatization of androgens to estrogens [46]; and stromal cells similar to fibroblasts, which support the function of the organ while holding it together [47]. These cells contribute not only to the development of the ovarian follicles containing oocytes and somatic cells but also the release of the mature egg from the ovary and the generation of the corpus luteum following ovulation and fertilization [48].

Ovarian cancer is the second most common gynecological cancer with the second largest number of deaths from gynecological malignancies in women [49]. While it has long been accepted that ovarian cancer emerges from within the ovary, studies have confirmed that its origin frequently lies in the ovarian surface epithelium [50,51], thus separating tumors into two main categories: epithelial and non-epithelial. However, further investigation into its histological and clinical characteristics has led to subgrouping into four main types: type I and II epithelial ovarian cancer (EOC), sex cord-stromal tumors, and germ cell tumors (based on presumed histogenesis and direction of differentiation) [49,52], as summarized in Table 1. Type I epithelial ovarian tumors include clear cell, endometrioid, mucinous, squamous, transitional cell, and low-grade serous carcinomas, and are generally associated with a more favorable prognosis [53]. Type II epithelial ovarian cancers encompass mixed mesodermal tumors as well as high-grade serous and undifferentiated carcinomas, and are correlated with a more aggressive prognosis [54]. Hormonal status is known to influence the evolution of ovarian cancer. For instance, parity and oral contraception intake act as protective factors against ovarian cancer, meanwhile nulliparity and older age can increase the frequency of ovarian cancer as well as its aggressiveness [55,56]. A lower risk in developing ovarian cancer is observed in Asian women, who also present a better clinical outcome than Caucasian women [57]. However, in most cases, the prognosis of ovarian cancer is poor due to late diagnosis. At the time of diagnosis, peritoneal metastasis is usually present. A lack of relevant clinical prognostic markers for ovarian cancer further impedes the prognosis process, making it clear that further studies are required to optimize the therapeutic approach and overall survival rates.

## 3. Intracellular Calcium Regulation in Ovarian Physiology and Cancer

As a dynamic, multicompartmental, ever-changing organ, the ovary requires a series of extremely coordinated and complex events to take place in order to fulfill its elaborate functions. In recent decades, the development of analysis techniques and the interpretation of bioinformatics data have facilitated our understanding of the molecular mechanisms occurring in the cascade of events involved in the ovary functions. Numerous signaling pathways can be activated by different types of stimuli and modulated by cytosolic Ca^2+^ levels which, in turn, are dictated by various transmembrane transport proteins or intracytoplasmic proteins. The specificity of a signaling pathway for carrying out a certain process is achieved by creating complexes through the assembly of special proteins. For instance, granulosa cells are responsible, among others, for oocyte maturation, a process regulated by many factors, and it has been revealed that one of the most important physiological characteristics of this type of cell is the modulation of the intracellular concentration of Ca^2+^ ions [81].

Ca^2+^ is known to be actively involved in the regulation of various critical modulatory proteins, including enzymes, chaperone proteins, and transcription molecules, making its equilibrium one of the most essential preconditions of cell survival. To this extent, any uncontrolled increase and/or decrease in cytosolic and organellar calcium levels can lead to cellular damage and even cell death [82,83]. An increased influx of Ca^2+^ into the mitochondria involves depolarization of the mitochondrial potential, a system that facilitates the accumulation of reactive oxygen species (ROS) involved in cell senescence, including tumor cells. This accumulation of ROS precedes apoptosis, representing the main factor responsible for permeability transition pore (mPTP) opening [84]. Moreover, apoptosis in ovarian cancer cells can be induced by increasing the intracellular calcium concentration, whereas a deficit of intracellular calcium can cause redox imbalance, leading to damage of the intracellular membrane [85]. Therefore, loss in the balance of calcium levels in the cell [86,87] and between cellular organelles [84,88] is considered to a play significant role in the proliferation of ovarian cancer cells.

Cytosolic calcium levels vary either due to transient, repetitive spikes, or as a result of sustained calcium responses. Both the rate and magnitude of intracellular calcium spikes are involved in essential molecular processes such as gene expression [89] and enzyme activation [90]. While it has long been shown that a steady increase in the frequency of calcium oscillations may lead to a sustained rise in intracellular Ca^2+^ [91,92], the exact mechanism underlying the augmented frequency of Ca^2+^ transients is yet to be elucidated [93]. Furthermore, although calcium spikes have been described at length in electrically excitable cells such as muscle (smooth, skeletal, cardiac) or neuronal cells, the same cannot be said for epithelial cells. In non-excitable tissues, calcium signals are propagated amidst adjacent cells in the form of intercellular calcium waves, transferring information and thus facilitating the coordination of cellular behavior [94].

In ovarian cells, gonadotropic follicle-stimulating hormone (FSH) and luteinizing hormone (LH) not only increase cAMP production in order to initiate various biochemical processes, including sex steroid synthesis and cellular metabolism regulation, but they can also trigger cytosolic calcium elevation [95,96]. In granulosa cells, Flores et al. have demonstrated that LH induces a biphasic increase in intracellular calcium levels by intracellular store activation and transmembrane calcium influx, thus contributing to the future stages of cell differentiation [97]. On this basis, it has been proposed that inhibiting the oscillatory waves of Ca^2+^ should interfere with cell cycle progression in ovarian cancer. To this extent, Dziegielewska and colleagues have demonstrated that by inhibiting T-type Ca^2+^ channels with mibefradil, a T-type Ca^2+^ channel blocker previously used as an antihypertensive drug, proliferation of ovarian cancer cells decreased while apoptosis was enhanced, mainly due to the decline in the expression of the apoptosis inhibitor survivin. They further found that mibefradil also increased cell sensitivity to carboplatin [98]. Li and colleagues have recently shown that mibefradil also blocks Orai Ca^2+^ channels, thus reinforcing the interest to exploit this drug as an anticancer agent [99]. On a similar note, Lee et al. have tested four FDA-approved calcium channel blockers targeting ovarian cancer stem cells (CSCs). They found that these compounds not only decreased the expression of the antiapoptotic factors survivin, B-cell lymphoma 2 (Bcl-2), and myeloid cell leukemia 1 (Mcl-1), but also induced caspase activation, highlighting their potential use as anticancer agents [100].

The mechanisms involved in the progression of ovarian cancer through proliferation are multiple. One of the best known is the transfer of Ca^2+^ between the ER and mitochondria, which triggers the Krebs cycle, increasing ATP production and biosynthesis [101], thus facilitating autophagy and cell proliferation [84]. Calcium ion influx through the voltage-gated Ca^2+^ channel (VGCC) regulates DNA synthesis, transcription, volume regulation, secretion, and motility, thus explaining the central role of Ca^2+^ channels in the cancer process [86]. The microenvironment also has a strong impact on the survival and proliferation of ovarian tumor cells in both primary and metastatic sites, with calcium being able to provide the means by which the tumor microenvironment (TME) can signal cancer cells. An example of interaction between cancer and stromal cells in the metastatic niche can be observed in serous ovarian cancer: Ca^2+^ signals in ovarian cancer cells are induced by adipocytes, while Ca^2+^-dependent phosphorylation of the salt-inducible kinase 2 (SIK2) in ovarian cancer cells leads to the activation of fatty acid oxidation, AKT phosphorylation, and triggering of proliferative and pro-survival pathways [102]. In human ovarian cancer, it has been shown, in vitro, using HO8910 and A2780 cell lines, that T-type Ca^2+^ channel expression was significantly increased compared to normal ovarian tissues, and the inhibition of Ca^2+^ influx suppressed the proliferation of ovarian cancer cells while also leading to cycle arrest in G0/G1 phase [87].

### 3.1. Inositol Trisphosphate Receptors

Inositol trisphosphate receptors (IP_3_R) are members of the family of calcium release channels that are located in the membrane of the endoplasmic reticulum. They are activated and potentiated by several ligands (including inositol triphosphate, cytoplasmic calcium, adenophostin A, nucleotides, or ATP) and are ubiquitously expressed in different types of tissues [103]. There are three IP_3_R isoforms—IP_3_R type 1 (IP_3_R1), InsP_3_R type 2 (IP_3_R2), and IP_3_R type 3 (IP_3_R3)—expressed in mammals [104] in different amounts, with each isoform having the ability to form homo- and heterotetramers [105].

**Figure 2:**
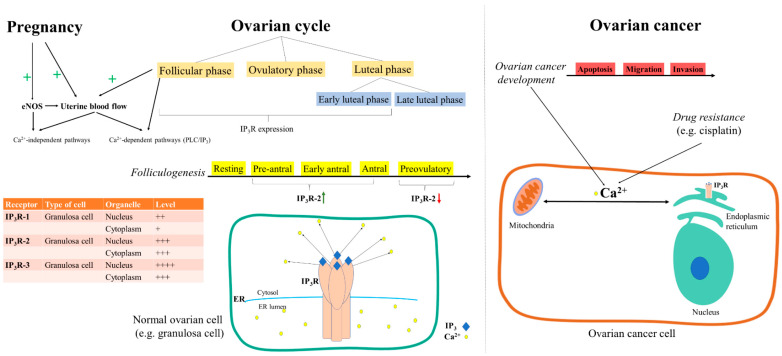
IP_3_R-mediated Ca^2+^ signaling in ovarian physiology and cancer. During pregnancy and the follicular phase of the ovarian cycle, uterine blood flow is increased, and this event is related to Ca^2+^-dependent (PLC/IP_3_) or -independent pathways [106]. IP_3_Rs are expressed in the follicular phase and the early luteal phase, but not in the corpus luteum in the mid-luteal phase of the ovarian cycle [109]. Granulosa cells express all IP_3_R subtypes, with distinct levels of expression (+, ++, +++, ++++) in nucleus and cytoplasm [81]. During folliculogenesis, IP_3_R-2 in granulosa cells are upregulated (↑) in the pre-antral and mid-antral phases, and downregulated (↓) in the preovulatory phase [109]. In ovarian cancer, the alteration of homeostasis in the ER–mitochondrial IP_3_R-mediated Ca^2+^ transfer is essential, contributing to ovarian cancer progression (apoptosis, migration, and invasion) and development of drug resistance [110,111,112,113,114].

Uterine blood flow is increased in pregnancy and during the follicular phase of the ovarian cycle (Figure 2). In this context, it is interesting to understand the intracellular calcium release pathways in the endothelium of the uterine arteries. It has been demonstrated in uterine artery endothelium isolated from pregnant sheep that 2-aminoethoxydiphenyl borate (2-APB, an IP_3_R antagonist) completely blocks ATP-induced intracellular calcium release, thus showing that ATP increases cytosolic calcium levels through the PLC/IP3 pathway while partly inhibiting ATP-induced NO release [106]. On the other hand, the 2-APB effect was tested in the presence of ionomycin, a calcium ionophore, and it was proven that 2-APB was unable to modify ionomycin-induced calcium release or NO production [106]. Yi and collaborators concluded that the increased activation of endothelial nitric oxide synthase (eNOS) in pregnancy is mediated through calcium-independent pathways [106].

Ovarian hormones promote folliculogenesis by modulating the local molecule synthesis that regulates cell contact between theca and granulosa cells in the ovarian follicle. In turn, theca cells produce hepatocyte growth factor (HGF) that can stimulate granulosa cell growth and, therefore, HGF is considered to play an important role in ovarian follicular development [107]. In rat ovarian surface epithelial cells, it has been demonstrated that HGF damaged cell contact while increasing IP_3_R3 expression, intracellular calcium levels, and apoptosis [108]. Meanwhile, the same study demonstrated that the expression of type 3 IP_3_R was increased when ovarian surface epithelial cells were grown in the absence of extracellular calcium [108], probably due to the loss of contact between cells. Moreover, granulosa cells have been shown to express IP_3_R types 1, 2, and 3 in endomembranes, the nuclear envelope, and intranuclear structures (Figure 2). It has also been demonstrated that IP_3_R are involved in the mobilization of cytoplasmic and nuclear Ca^2+^ in granulosa cells, and that the application of xestospongin, a non-competitive antagonist of IP_3_R (5 μM, 15 min), inhibited the ATP-mediated Ca^2+^ mobilization in both compartments, while basal Ca^2+^ remained constant [81].

The estrous cycle has also been demonstrated to regulate the expression of IP_3_R (Figure 2). More precisely, the expression of IP_3_R2 in porcine granulosa cells was studied during different phases of the estrous cycle, and it was found to be upregulated from the pre-antral stage, which is the first phase of folliculogenesis, when growth and differentiation of the oocyte takes place, to the mid-antral stage, when the oocyte completes its growth. Further on, IP_3_R2 was downregulated in preovulatory follicles, prompting the authors to conclude that it played a key role in the initiation and propagation of intracellular Ca^2+^ signals during follicular development [109].

Alterations of calcium homeostasis in ER and mitochondria have been shown to be involved in the resistance of ovarian cancer cells to chemotherapeutic drugs (e.g., cisplatin), especially since the ER–mitochondrial Ca^2+^ signaling pathway significantly contributes to cisplatin-induced cell apoptosis [110]. Moreover, there are certain cellular processes thought of as cancer hallmarks, including the onset of apoptosis, the emergence of drug resistance, and migration and invasion (both specific for metastasizing) that are greatly influenced by Ca^2+^ fluxes between the ER and mitochondria [111] (Figure 2).

All three isoforms of the IP_3_R were detected in ovarian tissue in granulosa cells in experimental organisms [81] and A2780 ovarian cancer cells [105]. Still, recent studies have found that IP_3_R3 has particularly been implicated in the prevention of neoplasia through pro-apoptotic mitochondrial transfer of Ca^2+^ ions. This process would be facilitated by the strategic position of these receptors, which are located within the mitochondria-associated ER membranes (MAMs) [112]. The release of Ca^2+^ ions through IP_3_R receptors is regulated by post-translational modifications related to the binding of Ca^2+^ ions to inositol triphosphate, coupling to IP_3_R receptors, the phosphorylation/dephosphorylation process, and the spatial distribution of ions in the cytoplasm [113]. By adjusting the transfer of Ca^2+^ from the ER to mitochondria, IP_3_Rs play a key role in cell survival/death. Increasing amounts of Ca^2+^ affect mitochondrial membrane integrity, leading to apoptotic cell death [114].

Moreover, IP_3_Rs also control cellular metabolism by supplying the mitochondria with Ca^2+^ ions, resulting in stimulation of the production of reducing equivalents through tricarboxylic acid (TCA) cycle-dependent enzymes involved in respiratory chain reactions to promote oxidative phosphorylation (OXPHOS) and ATP production. One of the distinguishing features of tumor cells is their ability to reprogram metabolism, which is of particular importance for the metabolic pathways that allow the continuous supply of metabolic intermediates resulting from the TCA cycle which are necessary for the proliferation of cancer cells [111,113]. It should also be noted that intracellular calcium levels are rigorously controlled by ER transport channels and pumps. High concentrations of Ca^2+^ from the lumen of the ER can migrate into the cytosol when the IP_3_R and RyR calcium channels open. In order to induce a concentration gradient, the ATP-dependent sarco-/endoplasmic reticulum Ca^2+^-ATPase (SERCA) pump located in the ER membrane needs to transport Ca^2+^ from the cytosol into the ER lumen. A recent study has shown that the basal hypothalamic–pituitary islands (BHPIs) trigger a continuous IP_3_R-dependent increase in cytosol calcium levels in ovarian cancer cells. Since IP_3_R Ca^2+^ channels remained open after BHPI treatment, the Ca^2+^ pumped into the ER rapidly leaked back out [115].

### 3.2. Ryanodine Receptors

**Figure 3:**
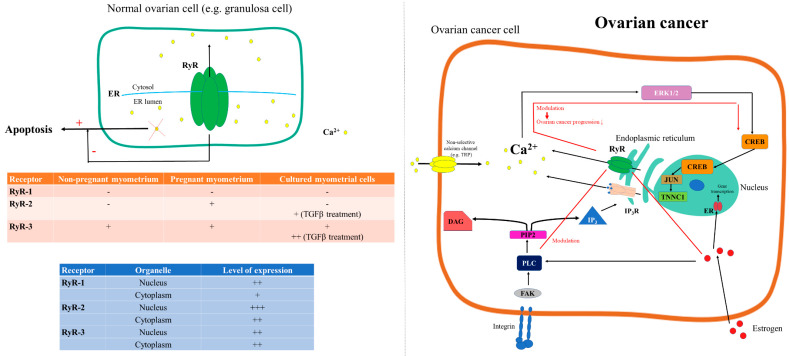
RyR-mediated Ca^2+^ signaling in ovarian physiology and cancer. RyR1 expression was not evidenced in either non-pregnant and pregnant myometrium nor in cultured myometrial cells. Meanwhile, RyR2 are expressed only in pregnant myometrium, and RyR3 are expressed both in non-pregnant and pregnant myometrium. Cultured myometrial cells express only RyR3, and TGF-β treatment upregulates both RyR2 and RyR3. Distinct subcellular expression of RyR subtypes was evidenced [81]. Persistent depletion of the ER Ca^2+^ deposits promotes apoptosis (+), while RyR inhibits (−) apoptotic cell death [122]. In ovarian cancer, RyR may contribute to the reduction of cancer progression by modulating the FAK//CREB/TNNC1 signaling pathway [127] and may modulate the ERα–PLCγ–IP_3_R pathway [128] (red line).

Díaz-Muñoz and colleagues described the presence of ryanodine receptors (RyR) in the endomembranes, nuclear envelope, and intranuclear structures of granulosa cells, with distinct levels of expression for each RyR isoform [81] (Figure 3). Ryanodine receptors are members of the family of calcium release channels that are located in the membrane of the endoplasmic reticulum and are ubiquitously expressed in a variety of tissues [116]. They are coupled to ion channels that are embedded in the inner part of the sarcoplasmic reticulum (SR) in the region where Ca^2+^ ions are stored, mediating its release from an intracellular membrane compartment and, thus, leading to the generation of a quick, transient increase in cytosolic calcium levels [117]. Ryanodine receptors are known to play key roles in the control of some major biological processes such as metabolism, cell–cell and cell–extracellular matrix relationships, proliferation and cell apoptosis, as well as cellular responses to different extracellular messages [118].

In ovary cells, Bhat and colleagues studied the expression of ryanodine receptors 1 and 2 (RyR1, RyR2) and evaluated their functions by recording the single-channel current and measuring intracellular Ca^2+^ using confocal microscopy [119,120]. They found that although ovarian ryanodine receptors could act as release channels both in vivo (caffeine-induced Ca^2+^ release) and in vitro (single-channel patch clamp experiments), they were, by themselves, insufficient for maintaining Ca^2+^ sparks comparable to those in muscle cells. Still, the expression and binding activity of ovarian RyR has been shown to significantly increase after dexamethasone exposure, reaching a peak 30 h following dexamethasone addition [121].

Similarly, in wanting to evaluate the effects that changes in intracellular Ca^2+^ homeostasis had on the apoptosis signaling pathway, Pan et al. found that stable RyR expression facilitated quick, reversible changes in cytosolic and ER Ca^2+^ loads by activating the RyR Ca^2+^ release channel with caffeine and ryanodine. Moreover, they revealed that persistent depletion of the ER Ca^2+^ deposits promoted apoptosis (Figure 3), while co-expression of B-cell lymphoma-extra-large (Bcl-xL) protein and RyR in these cells inhibited apoptotic cell death but no other forms of cell death [122].

The differential expression of ryanodine receptor mRNA has been studied in non-pregnant and pregnant human myometrium as well as in isolated cultured myometrial cells, where Awad et al. demonstrated the presence of the RyR2 and RyR3 isoforms, but not the RyR1 isoform (Figure 3). Moreover, treatment with the cytokine transforming growth factor beta (TGF- β) upregulated RyR2 and RyR3 in isolated cultured myometrial cells [123].

Ryanodine receptors can be involved in ovarian cancer through several mechanisms (Figure 3). They may intervene in multiple processes, such as cell resistance to calcium-induced apoptosis through the modulation of the Glutathione S-transferase omega (*GSTO*) gene [124,125], inhibition of the gonadotropin-induced extracellular signal-regulated kinase (ERK) 1/2 phosphorylation [126] and overall ovarian cancer progression by modulating the calcium-dependent FAK/CREB/TNNC1 signaling pathway [127]. However, RyR may also be involved in the activity of the estrogen receptor α (ERα) biomodulator, which may interfere, in ovarian cancer cells, in the ERα-PLCγ-IP_3_R pathway [128]. Furthermore, RyR have also been theorized to influence cells’ sensitivity to paclitaxel and doxorubicin and to control the activation of the unfolded protein response (UPR) in OVCAR-3 ovarian carcinoma cells [115].

### 3.3. Transient Receptor Potential Channels and Calcium Release-Activated Channels

Cellular Ca^2+^ entry is mediated both by transient receptor potential (TRP) proteins, which are nonselective cation channels permeant to Ca^2+^ [129], and calcium release-activated channels (CRAC), which are made up of a hexameric arrangement of Orai subunits surrounding a central ion-conducting pore [130]. The activation of these channels conducts cation influx, membrane depolarization, and the initiation of Ca^2+^-dependent signaling pathways.

TRP channels have been studied for decades and described as polymodal sensors that play various roles as cellular sensors and effectors for a large number of stimuli. The role of TRP channels has been demonstrated through various studies that discovered their involvement in physiological processes such as cell proliferation and migration, sensory processing, homeostasis and motile functions as well as fertilization. These roles are covered by a large family of TRP channels that have categorized, depending on their amino acid sequence homology, into seven groups: TRPC (canonical), TRPV (vanilloid), TRPM (melastatin), TRPP (polycystin), TRPML (mucolipin), TRPA (ankyrin), and TRPN (no-mechanoreceptor potential channel C) [131]. While the first six groups, amounting to 28 members, are found in mammals, the seventh group is present in insects, nematodes, fish, and amphibians. Mutations in the genes encoding TRP channels have been shown to result in various genetic disorders [132], which we have briefly summarized in Table 2.

In ovarian samples, research based on RNA extraction and synthesis of cDNA followed by PCR amplification have revealed the existence of TRP1 and TRP2 channels that play key roles in cellular Ca^2+^ homeostasis [133,134]. Furthermore, Gailly and colleagues found TRP2 channels to be involved in the compensatory calcium influx that occurs after the depletion of stores [134].

Vaca et al. have observed calcium release from the ER as well as calcium influx mediated by TRP1 channels and the influence of calmodulin and IP_3_R over the processes triggered by calcium load. Using confocal microscopy and electrophysiology measurements, they found a 900 ms delay between the release of calcium from the ER and the current through TRP1 channels. This delay was significantly increased after introduction of calmodulin into the cell, reaching approximately 10 s, demonstrating its inhibitory effect on channel activity [133].

CRAC channels have been demonstrated to act as key players of the immune system due to their ability to mediate calcium signaling in B and T cells as well as Fc receptors [135], with mutations in its subunit Orai1 leading to immune deficiency syndromes [136]. The activity of Orai and CRAC channels are dependent upon stromal interaction molecule (STIM), which is a calcium sensor located mainly in the ER that controls calcium store levels through its ability to connect to Orai subunits [137]. Increasing evidence has hinted that both TRP channels and Orai proteins are capable of shaping critical calcium-dependent mechanisms involved in the migration of stromal and cancer cells [138]. Moreover, they have been shown to cooperate not only with one another but also with other channels that are involved in cell migration [139], thus establishing their role in the spread of cancerous cells [140,141].

Transient receptor potential cation channels are expressed both in the plasma membranes and organelles of ovarian cancer cells. Recently, Liu et al. demonstrated a significant downregulation of TRPC1 in drug-resistant ovarian cancer tissues/cells [142]. The TRPC1 protein channel subgroup/subfamily has been found in many types of tissues, where it is involved in cell proliferation, differentiation, and migration, protection against cell death, functioning of smooth and skeletal muscle, etc., thus showing its involvement in physiological processes but also in regulating cancer evolution in various carcinomas, including ovarian cancer. Several studies have been developed in order to assess the contribution of TRPC1, however, little is known about its role in cell proliferation, tumorigenesis, and drug resistance in ovarian cancer. The interactions of TRPC1 with numerous proteins/genes, chemicals, biological processes, and mRNA, all involved in the regulation of ovarian cancer drug resistance and related to cell growth and death as well as gene expression, indicate a role for TRPC1 in drug resistance in ovarian cancer. TRPC mRNA expression shows decreased levels in human ovarian cancer cells vs. normal cells and marked downregulation in drug resistant vs. drug sensitive ovarian cancer cells [142].

The TRPC3 protein channel subfamily also seems involved in the evolution of ovarian cancer: it has been shown that a high expression of TRPC3 in human ovarian cancer cells enhanced the proliferation of ovarian cancer cells, while the inhibition of these channels resulted in growth suppression [143]. Ovarian cancer cells typically display high expression of TRPC3, be they functional or suppressed, because of the Ca^2+^ increase stimulated by EGF (epidermal growth factor). As TRPC3 is inhibited, ovarian cancer cell growth is suppressed. The epidermal growth factor receptor (EGFR) is critical for proliferation and tumorigenesis in ovarian cancer, with it being reported that EGF activates TRPC3/4/5, thus prompting Ca^2+^ inflow in HEK293 cells (human embryonic kidney 293 cells) [144,145,146,147].

Orai1/STIM1 have been shown to be upregulated in ovarian cancer cells resistant to chemotherapy. In their study, Schmidt et al. showed that the overexpression of Orai1/STIM1 occurred as a result of increased Akt1 activity in A2780 ovarian cancer cells [148]. Similarly, other authors have reiterated the anti-apoptotic effect of the store-operated calcium entry mediated by the STIM1/Orai1 complex [149,150], thus highlighting the need for updated treatment regimens that include platins combined with Akt1 or Orai1 inhibitors.

### 3.4. Calcium ATPases

Ca^2+^-ATPases are pivotal to the normal functioning of the ovary. In particular, these pumps play key roles in reproduction (e.g., through the follicles) and in production/binding of several types of hormones, such as luteinizing hormone (LH), follicle-stimulating hormone (FSH), estrogen, progesterone and androgens. Ca^2+^-ATPases intervene in the signaling between FSH and LH receptors by catalyzing the transformation of ATP into cAMP; cAMP, in turn, is an important messenger in this signaling pathway due to its ability to activate protein kinase A (PKA), which is further involved in modulating the expression of the LH receptor [151].

Depending on their location, Ca^2+^-ATPases are divided into three categories: sarco-/endoplasmic reticulum Ca^2+^-ATPase (SERCA), plasma membrane Ca^2+^-ATPase (PMCA), and secretory pathway Ca^2+^-ATPase (SPCA) [40]. Transmembrane and coiled-coil domain 1 (TMCO1), recently discovered proteins of the ER, are strongly involved in the proper functioning of the ovary, but are also closely related to Ca^2+^-ATPases due to their involvement in Ca^2+^ storage within the ER. TMCO1 can be found in the ER membrane, where it contributes to Ca^2+^ elimination when the ER is overloaded. TMCO1 loss of function has been connected to the reduction of ovarian follicles, as reported in studies conducted on mice [152]. The link between ER and TMCO1 Ca^2+^-ATPases is governed by SERCA pumps, tasked with transporting calcium ions from cytoplasm into the sarcoplasmic reticulum (SR) while also having catalytic properties regarding ATP [153]. The endoplasmic reticulum remains the main organelle that stores Ca^2+^ while also folding and assembling transmembrane proteins before their secretion. One important matter occurs when Ca^2+^ regulation is impaired, which leads to ER stress, thus hindering ER’s capacity to fold proteins. During the growth of the ovarian follicle, hypoxic conditions may lead to ER stress, therefore contributing to various pathological conditions in the ovaries, including ovarian cancer, polycystic ovarian syndrome, and ovarian hyperstimulation syndrome [154].

The three types of ATPases communicate with each other in different circumstances. For instance, both SERCA and PMCA pumps are involved in the restoration of basal Ca^2+^ levels. While SERCA pumps play a dual role in refilling the ER with Ca^2+^ and helping to switch off Ca^2+^ signaling, PMCA deals with Ca^2+^ passage through the plasma membrane [88,155]. PMCA is also involved in the survival of granulosa cells in the ovary as it mediates the ability of basic fibroblast growth factor (bFGF) to increase calcium efflux, and with its subsequent decrease, the number of apoptotic granulosa cells decreases [156]. PMCAs are responsible for eliminating cytosolic calcium surplus, thus managing to maintain optimum intracellular concentrations [157]. Their increased expression has been regarded as an indicator in the treatment of ovarian cancer: increased PMCA1 expression was found in ovarian cancer cells resistant to cisplatin, whereas its expression was lower in cells sensitive to this drug [158,159].

In the regulation of intracellular processes, ionized Ca^2+^ plays a pivotal role. Cell proliferation, apoptosis, motility, secretion, and tumor growth, among others, are events regulated by ionized Ca^2+^ acting as a universal second messenger. In apoptosis regulation, the influence of Ca^2+^ can be explained through the strong relationship between calcium homeostasis and members of the Bcl-2 family, known to possess both pro- (e.g., Bax and Bak) and anti-apoptotic (e.g., Bcl-2, Bcl-xL) properties [160]. Calcium entering the endoplasmic reticulum leads to a decrease in the amplitude of calcium signaling, thus preventing the triggering of apoptosis. Furthermore, calcium signals can modulate the expression of Bcl-2 proteins by activating the calcium/cAMP-responsive-element-binding proteins (CREBs) [161,162].

Alteration of calcium pumps and channels has been observed in cancer, which have an impact on cellular proliferation by activating survival pathways or preventing apoptosis [24]. The plasma membrane Ca^2+^–Mg^2+^-ATPase plays a crucial role in keeping the homeostasis of intracellular Ca^2+^. In advanced cases of ovarian cancer, Barylyak et al. have shown that the activity of lymphocyte plasma membrane Ca^2+^–Mg^2+^-ATPases were considerably dissimilar to the physiological standard, showing decreases by 1.6 and 1.8 times, which they linked to the rise of cytosolic Ca^2+^ in blood lymphocytes [163]. Further on, SERCA pumps, although normally involved in physiological events, may undergo mutations leading to their increased expression, which has been observed in ovarian cancer, thus suggesting the contribution of SERCA in its development. Due to the increase in SERCA expression levels, changes in ovarian cell Ca^2+^ levels have also been reported [164]. Moreover, PMCA1 has been revealed to be involved in ovarian cancer, inducing cisplatin resistance. This observation has been reported in a study using A2780 human ovarian cancer cell lines, which suggested that the alteration in calcium homeostasis maintained by PMCA1 led to the development of platinum-resistant ovarian cancer phenotype [158].

Other studies have suggested that ATPases may be useful in the treatment of ovarian cancer, in combination with conventional treatments such as platinum-based antineoplastic agents [165] or tumor radiation therapy [166]. However, in certain situations, calcium ATPases may induce resistance to commonly used chemotherapy drugs, such as cisplatin. For instance, Al-Bhalani et al. have demonstrated that, when interacting with *TP73*, Ca^2+^-ATPases can induce either resistance or sensitivity to cisplatin, depending on the regulation of the gene in question and on the action of the ATPase [167]. Calcium is involved in cell progression under physiological conditions, but it is evident that it can also intervene in uncontrolled cell proliferation, leading to tumorigenesis. Drug targeting of calcium channels may therefore help put an end to uncontrolled proliferation [87].

### 3.5. Mitochondrial Calcium Channels

Mitochondria are vital double-membrane organelles with a crucial role in providing energy, thus assuring the survival and thriving of cells. In the oocyte, the mitochondrion acts as a source or supply of adenosine triphosphate (ATP) during fertilization and preimplantation development while also storing calcium along with various pro-apoptotic factors [168,169]. Apart from that, however, these organelles guard their own genetic material, originating from maternal DNA, namely mitochondrial DNA (mtDNA). Together with the smooth ER, mitochondria form complex structures that join forces to ensure both the production and storage of the necessary ATP for fertilization [168]. Furthermore, the intracellular Ca^2+^ required for the maturation of the oocyte is also provided by these aggregates [170]. Calcium homeostasis in the mitochondrion is primordially ensured by the mitochondrial calcium uniporter (MCU), a transmembrane protein that facilitates Ca^2+^ transport within its lumen, by making use of the inner mitochondrial membrane (IMM) negative charge [171]. MCU is part of a larger, more complex structure consisting of MCU and its regulators, MCUb, making up the channel [172]; mitochondrial calcium uptake proteins 1 and 2 (MICU1 and MICU2); and the essential MCU regulator (EMRE) [173]. MICU1 has been shown to have a stimulating effect on MCU activity, while MICU2 directly suppresses it, acting together as a regulatory dimer made up of two subunits with opposite functions [172]. However, at low cytosolic Ca^2+^ levels, MICU1 can also have an inhibitory role [174].

Importing Ca^2+^ into the mitochondrion is necessary not only for the overall maintenance of intracellular calcium homeostasis, but also for oxidative phosphorylation so as to produce ATP [175]. However, it has been demonstrated that excessive Ca^2+^ intake is causally related to both replicative and oncogene-induced senescence [176,177]. Silencing of MICU1 leads to the abolition of MICU2, but not the other way around, rendering the uniporter ineffective [178,179] while also evading oncogene-induced senescence [180]. Chakraborty et al. have recently studied MICU1 expression in both normal and cancerous ovarian cells and found it to be either missing or minimally expressed in normal ovarian cells, its absence or low expression virtually acting as a cancer-protective factor [181].

Proto-oncogenes and tumor suppressor genes can act, at the mitochondrial level, as a response to a set of stressful stimuli by regulating the MCU complex, thus adjusting mitochondrial Ca^2+^ concentration. Studies performed on CP20, A2780, OSE, OV90, OV1487, OVCAR4, OVCAR2, and SKOV3 cell lines have shown that overexpressed MICU1 is responsible for deranged cell metabolism and drove aerobic glycolysis in ovarian cancer cells, thus playing an important role in the poor outcome of ovarian cancer due to chemoresistance [181]. Similarly, Arvizo and colleagues have recently shown that MICU1 promotes the resistance of ovarian tumor cells to positively charged gold nanoparticles by sequestering Ca^2+^ within its lumen and avoiding cell death [182]. MICU1 could therefore become an important therapeutic target for the normalization of mitochondrial oxidative metabolism, leading to a restoration of sensitivity to chemotherapy.

Drug resistance in tumor cells can therefore be attributed to the decreased apoptosis that appears as a result of mitochondrial Ca^2+^ accumulation and increased mitochondrial membrane permeabilization [183,184]. Moreover, the decrease in ER Ca^2+^ content is linked to diminished apoptosis followed by survival and proliferation of tumoral cells [185]. Metastasis and invasion have also been reported to be directly correlated to the mitochondrial Ca^2+^ uniporter and mitochondrial Ca^2+^ linked to hypoxia-inducible factor 1 (HIF1α) signaling, with functions in metabolic reprogramming, metastasis, and invasion [84].

### 3.6. G-Protein-Coupled Receptors

G-protein-coupled receptors (GPCRs) are a family of proteins consisting of more than 800 members identified in the human genome. The conformation of GPCRs consists of seven transmembrane spanning α-helices linked by three intracellular and three extracellular loop regions. They also have an extracellular amino-terminal domain and an intracellular carboxyl tail [186]. GPCRs distinguish themselves, in this case, by binding extracellular stimuli, which activates G-proteins, thus triggering cascade responses at cytoplasmic and nuclear level [187]. Heterotrimeric guanine-nucleotide-binding regulatory proteins (G-proteins) are made up of three subunits―α, β, and γ―which, by binding to a ligand, release guanosine diphosphate (GDP), replacing it with guanosine triphosphate (GTP) [186]. Ligands that can be associated with GPCRs are numerous, starting from biogenic amines, such as noradrenaline, dopamine, histamine, and acetylcholine, to amino acids and ions (glutamate, calcium, γ-amino butyric acid), lipids (prostaglandins, leukotrienes, sphingosine-1- phosphate), peptides and proteins (chemokines, angiotensin, thrombin, bombesin, endothelin, bradykinin), odorants, nucleotides, cannabinoids, endorphins, opiates, pheromones, as well as physical stimuli such as light [188,189]. Following the binding of the agonist, the conformation of the receptor changes as a result of the dissociation of GDP and its substitution with GTP, dividing G-protein complexes into α subunits and βγ dimers, each of them producing different effects [190].

The α subunits can be classified into four types―Gαs, Gαi, Gαq, and Gα12―which, along with the βγ dimers, can activate several effectors. To this extent, they can either stimulate or inhibit adenylyl cyclase, increase or decrease cAMP levels, activate phospholipase C (PLC), stimulate small GTP-binding proteins of the Ras and Rho families, activate MAPK family members, as well as stimulate ion channels and lipid kinases [188]. These processes contribute to the regulation of gene expression [191], with second messenger responses triggering a cascade of biological processes such as angiogenesis, cancer progression, cell survival, differentiation, and proliferation [189].

GPCRs have been shown to be involved in tumorigenesis and metastasis, with several studies reporting the implications of the coupling of steroid hormones with GPCRs in ovarian cancer [192,193]. Moreover, ovarian cancer may also be associated with altered signals in the nervous system and immune system, as well as various inflammatory states [194,195]. These signals can be mediated by GPCRs, contributing to the tumorigenesis process. Such signals can be induced by muscarinic, adrenergic, serotoninergic, dopaminergic, bradykinine, histamine, and chemokine receptors, many of which mediate Ca^2+^ signaling. Although these mechanisms have not been extensively studied, they provide promising leads for future perspectives regarding ovarian cancer management [41]. The role of Ca^2+^ transients via GPCRs in ovarian cancer has been investigated in several studies analyzing the effects of various stimuli on ovarian cancer cell lines, as summarized in Table 3.

GPCRs have also been regarded as drug targets, since their activity has been estimated to be influenced by over 25% of the drugs accepted by the Food and Drug Administration [199]. Therefore, understanding the exact roles of GPCRs in ovarian cancer is essential for the development of new ovarian cancer therapies [192].

### 3.7. Hormone Receptors

The most important part of the ovary is the follicle, regardless of its development stage. Follicle growth and development are regulated and maintained by the gonadotropins follicle-stimulating hormone (FSH) and luteinizing hormone (LH) [151]. FSH and LH are part of the glycoprotein hormone family, alongside the human chorionic gonadotropin (hCG) and the thyroid-stimulating hormone (TSH). They express a common alpha subunit but are differentiated through the uniqueness of their beta chains. The effect of FSH is visible after its binding to the FSH receptor (FSHR), located on granulosa and Sertoli cells [200]. FSHRs belong to the family of G-protein-coupled receptors (GPCRs) consisting of seven transmembrane domains: three extracellular loops, three short intracellular loops, and one intracellular tail. They also have 340–420 amino acid-long ectodomains that are able to bind ligands with high molecular masses [201,202].

After binding to their respective receptors, FSH and LH increase the production of cyclic adenosine monophosphate (cAMP), resulting in greater estradiol production. The pathways by which the two receptors act are quite similar, the main difference between them being that while FSH receptors are only found in granulosa cells, LH receptors reside in the theca interstitial cells. However, the process occurs similarly, as the increase in cAMP level activates the production of estradiol, which is then released into the bloodstream and follicular fluid within the follicular antrum [151,202]. Estrogens tend to induce changes through different pathways, however, the most important is the interaction with the estrogen receptor (ESR). ESR is localized in the nucleus or plasma membrane, and classified into two subtypes: ESR alpha and ESR beta, encoded by two different genes: *ESR1* and *ESR2* respectively. These receptors belong to the nuclear receptor superfamily, having structural domains from A to F. The D-domain, in particular, plays an important role, as it interacts with the activator protein 1 (AP1), generating fluctuations in mRNA levels as well as distinct physiological responses in a process that takes up to several hours. However, when estrogen acts at an ESR level in the plasma membrane, and not at nuclear level, with cellular response increasing Ca^2+^ concentrations, the process is shortened to only a few seconds [203,204].

Alongside estrogen, another important hormone involved in the normal functioning of the ovaries is progesterone, which is produced similarly to estradiol. Progesterone binds to the progesterone receptor (PR), a protein expressed in two isoforms, PR-A and PR-B, which are transcribed from the same gene. Their task is to regulate the transcription of progesterone-sensitive genes [205]. While PR-B tends to perform this function by activating these genes, PR-A intervenes in their control as a repressor of PR-B, also decreasing the responsivity to other hormones, such as estrogen or androgens [206].

A vast amount of work has studied the involvement of androgen receptors (ARs), estrogen receptor alpha (ESRα), and progesterone receptors (PRs) in the pathophysiology of ovarian cancer, with a particular interest in patient survival. Sexual steroid hormones acting through their receptors activate signaling pathways that play key roles in tumor evolution. These pathways are related to cell proliferation, migration, tumor invasiveness, epithelial–mesenchymal transition, and apoptosis [207,208,209,210]. Postmenopausal hormone replacement therapy (HRT) with estrogen for a period of 10 years or longer revealed the effect of estrogen in ovarian cell proliferation, showing an increased risk of ovarian cancer generated from the constant exposure of the ovarian surface epithelium to estrogen [55]. Moreover, the use of hormones as treatment for ovarian cancer is not widely recommended [211]. Patients with ovarian cancer record high levels of estrogen, which increases the mobility of cancer cells by impairing cell adhesion and facilitating metastasis. The effects of estrogen and progesterone on the proliferation and apoptosis of ovarian cancer cells are rendered possible through ESRs and PRs [212]. Moreover, ESR/PR positivity in ovarian cancer has been associated with early peritoneal metastasis with high recurrence rate [213]. HGSC is characterized by a high frequency of both triple-negative and AR+/ER−/PR+ profiles, while endometrioid carcinoma is associated with triple-positivity at a higher frequency [214]. No difference has been recorded in the frequency of ESR or PR positivity in any of the four subtypes of epithelial ovarian cancer between pre- and postmenopausal patients, with the exception of serous carcinomas, where PR positivity was significantly higher in premenopausal than postmenopausal women [215,216]. Several studies have shown that ESR/PR positivity in ovarian cancer has an impact on prognosis and treatment response [217,218,219,220,221].

Steroid hormone receptors have characteristic profiles depending on the ovarian carcinoma subtypes. An inexpensive evaluation method of AR, ESR, and PR expression and co-expression alongside the proliferation index could be applied to patients with ovarian carcinoma and correlated with survival rates. The characterization of the steroid hormone profiles in ovarian carcinoma may conduct the drafting of personalized cures with less aggressive hormonal and anti-hormonal treatments.

## 4. Contribution of Altered Calcium Signaling to the Development of Chemotherapy Resistance in Ovarian Cancer

Over time, the standard management of ovarian cancer has shown some improvement regarding clinical response and survival rates, however, there are numerous cases which undergo relapse or are chemoresistant, thus determining the high mortality rates. First-line chemotherapy is still based on the combination of platinum-based anticancer drugs and taxanes, such as paclitaxel, for women with advanced ovarian cancer. After applying this protocol, the platinum-free interval (PFI) is monitored as the interval between the date of the last dose and the date of relapse detection [222]. Patients in advanced stages can also benefit from treatment with antiangiogenic agents such as bevacizumab, either added to the chemotherapy regimen or administered after chemotherapy discontinuation [223]. Despite all efforts, relapse is found in 25% of patients with early stage disease and over 80% of patients with advanced disease [224]. Therefore, there is a dire need for development of new strategies to overcome drug resistance.

Various therapeutic drugs used in oncology perform their action by inducing the mobilization of Ca^2+^ stores, thus triggering apoptosis through the transient elevation of intracellular free calcium levels [225]. Platinum-based antineoplastic drugs act through interaction with nuclear DNA-related genes [101] while also accumulating in different organelles, including the endoplasmic reticulum, lysosomes, and mitochondria, triggering apoptosis [226,227]. Kucukkaya et al. showed that fifteen genes associated with calcium homeostasis (*IP_3_R1-3, RYR1/2, SERCA1-3, NCX1-3, PMCA1-4*) were downregulated in cisplatin resistant cells, thus suggesting the use of regulators of these genes as a potential therapeutic strategy [228]. Büsselberg and Florea proposed the hypothesis that intracellular calcium signaling influences the expression of the genes responsible for multidrug resistance [225]. In their opinion, gene expression can also be modulated by the action of epigenetic factors, which can also modify intracellular calcium signaling. In this way, protein expression is modified in multidrug resistant cancerous cells [229]. Xie et al. demonstrated that the pharmacological inhibition of Bcl-2 through ABT737 or genetic knockdown of Bcl-2 reversed the resistance of SKOV3/DDP cells to cisplatin therapy by augmenting cytoplasmic and mitochondrial Ca^2+^ levels [230]. Along the same lines, the repurposing of auranofin, an antiarthritic drug, for anticancer therapy, has also been proven to be effective due to its ability to elevate intracellular Ca^2+^ levels and thus induce apoptosis [231]. Marzo and colleagues then showed that replacing the thiosugar from auranofin with an iodide ligand yielded superior antineoplastic activity in an orthotopic ovarian cancer model [232].

Variations in both gene expression and regulation may have a significant impact on the outcome of anticancer therapies [233]. One study performed on MDAH-2774 epithelial ovarian cancer cell line and its chemoresistant subclone showed that the intracellular calcium concentration was decreased in cells resistant to cisplatin. Moreover, the mRNA expression profiles for genes responsible for calcium regulation (e.g., *SERCA1/2/3, IP_3_R1/2/3, RYR1/2, PMCA1/2/3*) were decreased in the cisplatin resistant cell line compared to those from parental cells [228].

Tumor chemoresistance, either intrinsic or acquired, might be improved if T-type Ca^2+^ channels known to affect ovarian tumor growth and response to platinum agents were targeted. Inhibition of T-type Ca^2+^ channels with mibefradil and simultaneous administration of carboplatin rendered platinum-resistant ovarian tumors sensitive and increased cancerous cell apoptosis [87,98]. Furthermore, the activation of the calcium-dependent potassium channel KCa3.1 at the same time as the blockade of the voltage-gated potassium channel Kv11.1 via riluzole has been shown to be a promising therapeutic strategy for overcoming cisplatin resistance [234].

Bonnefond et al. analyzed the effect of multiple inhibitors of calcium signaling in ovarian cancer cell lines. Their aim was to detect whether pharmacological inhibition of calcium signaling was sufficient to decrease the expression of the anti-apoptotic Mcl-1 protein and, therefore, sensitize ovarian cancer cells to anti-Bcl-xL with antibodies directed towards the overexpressed anti-apoptotic protein Bcl-xL, which is generally associated with poor prognosis. Their results indicated that calcium signaling regulated Mcl-1, thus opening a new path for the use of calcium modulators to target Mcl-1, be it directly or indirectly, as a crucial step for successful chemotherapy [235].

## 5. Conclusions

It is now indisputable that calcium signaling, an efficient and versatile mechanism, plays a dual role in cell proliferation and apoptosis. On the one hand, this knowledge should incite great caution in the clinical setting when using calcium-modifying drugs, foods, and nutrients. On the other hand, numerous antineoplastic drugs rely on their ability to modulate intra- and extracellular calcium levels so as to trigger apoptosis, some of them even making use of calcium-chelating agents in order to enhance their activity. Therefore, there is a huge need for better understanding cellular mechanisms underlying the regulation of Ca^2+^ signaling in tumor cells. It is becoming evident that Ca^2+^ signaling is deeply involved in fundamental aspects of carcinogenesis, from initiation to recurrence and metastasis. The fact that Ca^2+^ reduction is, in a manner, linked to malignant cell growth restriction makes it quite imperative that individual calcium channels be more effectively identified. Moreover, while changes in the expression of channels and pumps have been described in a variety of cancer types, the need for a clear depiction of specific modifications that arise in the proteins in charge of Ca^2+^ regulation persists, as it might aid in deciphering and ideally controlling them, either through pharmacological or genetic means.

Admittedly, the ubiquity of Ca^2+^ signaling in both normal and cancer cells makes it burdensome to thoroughly investigate, which remains vital for the development of novel therapies. Restoring Ca^2+^ homeostasis and dynamics remains a promising path to follow, guiding us to a new understanding of therapeutic targeting. Novel therapeutic approaches targeting channels, receptors, or transporters involved in Ca^2+^ signaling should also be taken into consideration in curing cancer.

## Figures and Tables

**Table 1:** Ovarian cancer classification.

Cancer Type	Histological Subtype	Distinguishing Features
Type I epithelial ovarian cancers	Clear-cell carcinoma	- frequently associated with endometriosis [58]; - genetic mutations are responsible for excessive glycogen synthesis [59], resulting in cellular glycogen accumulation which, in turn, promotes cell growth [60].
	Endometrioid carcinoma	- frequently associated with atypical endometriosis [61]; - both ovaries may be affected in up to 50% of cases [62].
	Mucinous carcinoma	- tumors can reach impressive sizes due to the accumulation of a mucus-like substance secreted by goblet cells [63]; - *KRAS* mutations have been reported in 40–50% of cases [64].
	Squamous carcinoma	- a rare subtype of EOC, most often occurring as a malignant transformation of a mature cystic teratoma [65].
	Transitional cell carcinoma	- a rare subtype of EOC originating from pluripotent stem cells of the germinal epithelium and transitional urothelial cells [66].
	Low-grade serous carcinoma (LGSC)	- it associates relatively high ER and PR expressions, making endocrine therapy possible [67]; - when present, mutations in genes of the *KRAS–BRAF–MAPK* pathway can act as targets for anticancer therapy, thus leading to a positive effect on the overall survival rate [68].
Type II epithelial ovarian cancers	Mixed mesodermal tumor	- rare tumors, histologically associated with carcinomatous and sarcomatous features [69].
	Undifferentiated carcinoma	- associated with an aggressive clinical course and poor prognosis [70]; - poorly differentiated tumors that, although challenging to categorize histologically, are considered epithelial due to the presence of surface epithelial components [71].
	High-grade serous carcinoma (HGSC)	- the most frequent EOC subtype, accounting for up to 80% of ovarian cancer deaths [54,72]; - often diagnosed in advanced stages, making it difficult to establish its source. It appears to originate both in the ovary and commonly in the fallopian [73,74]; - *TP53* mutations are present in up to 97% of cases [75,76].
Germ cell tumors	Germ cell tumors	- risk factors include the use of exogenous hormones, teenage pregnancy, endometriosis [77], as well as genetic mutations (e.g., altering of the tumor suppressor gene *TRC8/RNF139*) [78].
Sex cord-stromal tumors	Sex cord-stromal tumors	- rare tumors arising from the tissue supporting the ovary, more often seen in young adults [79]; - due to the involvement of the constituent cells in hormone synthesis, these tumors can associate endocrine syndromes including hyperandrogenic/hyperestrogenic states [80].

**Table 2:** TRP channels and associated genetic disorders.

TRP Family	TRP Members	Associated Genetic Disorder
TRPC	TRPC1, mTRPC2, TRPC3/4/5/6/7	Focal segmental glomerulosclerosis (TRPC6)
TRPV	TRPV1/2/3/4/5/6	Olmsted syndrome (TRPV3)
		Type 3 brachyolmia (TRPV4)
		Hereditary motor and sensory neuropathy type 2 (TRPV4)
		Congenital distal spinal muscular atrophy (TRPV4)
		Spondyloepiphyseal dysplasia Maroteaux type (TRPV4)
TRPM	TRPM1/2/3/4/5/6/7/8	Autosomal recessive congenital stationary night blindness (TRPM1)
		Progressive familial heart block type IB (TRPM4)
		Primary hypomagnesemia with secondary hypocalcemia (TRPM6)
TRPML	TRPML1/2/3	Mucolipidosis type IV (TRPML1)
TRPP	TRPP1-PKD2, TRPP2-PKD2-L1, TRPP3-PKD2-L2	Autosomal dominant polycystic kidney disease (TRPP1-PKD2)
TRPA1	TRPA1	Familial episodic pain syndrome

**Table 3:** GPCRs involved in ovarian cancer and Ca^2+^ modulation.

GPCR	Stimuli	Calcium Effect	Type of Cells	Effect	Reference
Lipid Receptor	S1P1/3	Increased intracellular Ca^2+^	OVCAR-3 cells	Chemotactic migration Cellular invasion	Park et al. [196]
Lipid Receptor	LPE	PTX-sensitive G-protein-dependent Ca^2+^ increase	SKOV-3 cells OVOCAR 3 cells	Chemotactic migration	Park et al. [197]
Biogenic amines	ATP Histamine	Increased intracellular Ca^2+^	SKOV-3 cells	Cellular proliferation	Batra et al. [198]

S1P = sphingosine-1-phosphate receptor; LPE = lysophosphatidylethanolamine; PTX-sensitive G-protein = Pertussis toxin (PTX)-sensitive GTP-binding protein.
